# An Efficient Micro Control Unit with a Reconfigurable Filter Design for Wireless Body Sensor Networks (WBSNs)

**DOI:** 10.3390/s121216211

**Published:** 2012-11-22

**Authors:** Chiung-An Chen, Shih-Lun Chen, Hong-Yi Huang, Ching-Hsing Luo

**Affiliations:** 1 Instrumentation Chip Group, Department of Electric Engineering, National Cheng Kung University, Tainan 701, Taiwan; E-Mail: n2895159@mail.ncku.edu.tw; 2 Department of Electronic Engineering, Chung Yuan Christian University, Chung Li City 320, Taiwan; E-Mail: chrischen@cycu.edu.tw; 3 Graduate Institute of Electrical Engineering, National Taipei University, Taipei 10478, Taiwan; E-Mail: hyhuang@mail.ntpu.edu.tw

**Keywords:** micro control unit, reconfigurable filter, multi-sensor, asynchronous interface, wireless body sensor network

## Abstract

In this paper, a low-cost, low-power and high performance micro control unit (MCU) core is proposed for wireless body sensor networks (WBSNs). It consists of an asynchronous interface, a register bank, a reconfigurable filter, a slop-feature forecast, a lossless data encoder, an error correct coding (ECC) encoder, a UART interface, a power management (PWM), and a multi-sensor controller. To improve the system performance and expansion abilities, the asynchronous interface is added for handling signal exchanges between different clock domains. To eliminate the noise of various bio-signals, the reconfigurable filter is created to provide the functions of average, binomial and sharpen filters. The slop-feature forecast and the lossless data encoder is proposed to reduce the data of various biomedical signals for transmission. Furthermore, the ECC encoder is added to improve the reliability for the wireless transmission and the UART interface is employed the proposed design to be compatible with wireless devices. For long-term healthcare monitoring application, a power management technique is developed for reducing the power consumption of the WBSN system. In addition, the proposed design can be operated with four different bio-sensors simultaneously. The proposed design was successfully tested with a FPGA verification board. The VLSI architecture of this work contains 7.67-K gate counts and consumes the power of 5.8 mW or 1.9 mW at 100 MHz or 133 MHz processing rate using a TSMC 0.18 μm or 0.13 μm CMOS process. Compared with previous techniques, this design achieves higher performance, more functions, more flexibility and higher compatibility than other micro controller designs.

## 1. Introduction

A wireless body sensor network (WBSN) [1,2] consists of spatially distributed autonomous body sensors that are used to monitor physical signals, such as temperature, blood pressure, electrocardiogram (ECG), and heartbeat, and then transmit the information thus obtained through the wireless network to central servers. WBSN systems are helpful for healthcare monitoring applications. For example, for more than 25% of patients, the blood pressure measurements taken in a doctor’s office or hospital may be higher than usual, due to so-called white-coat hypertension. WBSN systems can monitor the blood pressure for 24 hours, thus avoiding this situation and providing more accurate information for diagnostic purposes. Although WBSN systems are widely used for long-term healthcare monitoring applications, their acceptance is limited by the power, size, and cost constrains of each sensor node in corresponding to limited resource of battery, memory, computational speed, and wireless communication bandwidth. It is thus important to develop low-power, low-cost, small-size, and high performance wireless body sensor nodes.

A typical wireless body sensor node is composed of the following parts: bio-sensors, a readout circuit, an analog to digital converter (ADC), a micro control unit (MCU), and a wireless transceiver with an antenna. Researchers have proposed a number of efficient bio-sensors for use with WBSN systems. For example, thermal sensors [3] are used to detect fever in babies and child mortality, while heartbeat and electrocardiogram (ECG) [4] sensors are used to prevent heart disease, and blood pressure (BP) [5] can be also measured to reduce the risk of some cardiovascular diseases. Recently, some special biomedical signals can be measured or detected by image process techniques through digital colour camera CCD-based sensors [6]. The microcontroller [7] with its functions of power management, signal processing, and interfacing between the ADC and transceiver is an important device in a WBSN system. Researchers have proposed a number of low-cost and high-performance microcontrollers [8–11]. However, while micro-control units [10,11] are designed as digital signal processors (DSPs), and have high performance with regard to processing signals, they also consume a huge amount of power. In our previous works [8,9], an adaptive power controller was designed into a micro control unit (MCU) and used to develop a low power WBSN system [9]. A low-cost VLSI architecture for a micro control unit [8] with multi-sensor nodes was also designed for a WBSN system, with the functions of data compression, asynchronous interface, ECC, and power management.

A digital filter [12] is a system that performs mathematical operations on a sampled signal to reduce or enhance certain aspects of the original signal. The low pass filters [13] pass signals from low frequencies and reject signals from high ones. Forecasting is the process of making statements about future events, and it can be useful for Fuzzy-Logic decision-making [14] to help make long-term energy purchases. The lossless data encoder used with a low pass filter can reduce the data rate without losing any information. Huffman coding [15,16] is an entropy encoding algorithm used for the proposed lossless data encoder design, while the error correct coding (ECC) [17] is a technique used to control errors during data transmission, which can improve the reliability of wireless communication.

As mentioned above, to improve the functions of a WBSN system, it is necessary to enhance the design of the MCU. In this paper, a versatile MCU design is proposed, consisting of an asynchronous interface, register array, reconfigurable filter, sloped-feature forecast, and lossless data encoder. The power management (PWM) and multi-selector systems are also designed for to support low-power use and many different sensors, respectively. Moreover, the universal asynchronous receiver/transmitter (UART) interface is designed to improve compatibility with wireless devices. The rest of this paper is organized as follows: Section 2 introduces the system architecture, while Section 3 presents the VLSI architecture of the proposed design. Section 4 shows the simulation results and chip implementation. Finally, Section 5 presents some brief conclusions to this work.

## 2. Architecture of WBSN System

**Figure 1.**
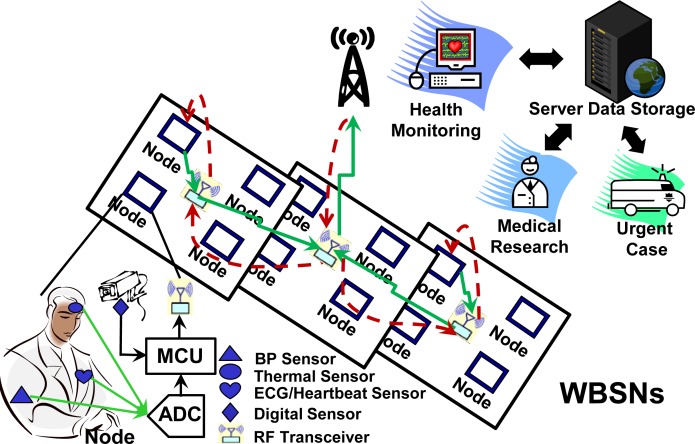
WBSN system for hospital and healthcare monitoring applications.

Figure 1 shows the architecture of the WBSN system for a hospital and healthcare monitoring application. In this case, each wireless body sensor node connects with various physical and physiological sensors. The hierarchical architecture of this WBSN system [9] assembles four nodes to a group layer, and combines several groups to a network layer. The network layer controls all of the sensor groups by sending commands and receiving data from them. Each wireless body sensor node is integrated by bio-sensors, analog-to-digital converter (ADC), MCU, and wireless transceiver module. The analog bio-signals are captured by bio-sensors and then converted from analog to digital type. If the bio-signals are from digital sensors, the detected signals can be sent to the MCU directly. Finally, the processed bio-signals are transmitted by wireless devices through the communication UART protocol. The information of bio-signals can be refined and displayed by a computer or server within a few seconds.

Different from a microprocessor that uses a sequential process, the MCU design can process data by parallel computing. The main advantage of parallel computing is that large operations can be divided into smaller modules to solve in parallel synchronously, which provides the needed high performance for real-time bio-signal processing. It also means that the operating frequency can be decreased for the same operations, leading to lower power consumption, which is a key characteristic for wireless devices. To communicate and synchronize signals between different subtasks, an asynchronous circuit is needed to maintain the high performance of the parallel program and correction of the transmission signals. The MCU device not only has the function of data processing, but also produces control signals that control each part of the wireless body sensor node.

## 3. VLSI Architecture of MCU

**Figure 2.**
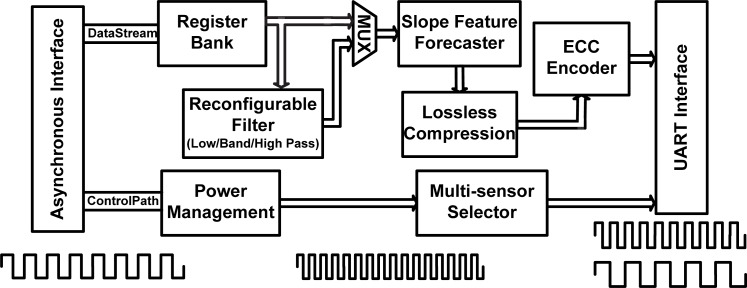
The block diagram of the proposed micro-control unit (MCU).

Figure 2 shows the block diagram of the proposed MCU design. It can be separated into the data and control paths. The control path carries out the controlled signal processing, and the data is transmitted via the data stream path. As Figure 2 shows, the proposed MCU design consists of an asynchronous interface, a power management circuit, a register bank, a reconfigurable filter, a slope-feature forecaster, a lossless compression circuit, a multi-sensor selector, an ECC encoder, and a UART interface. The details of each part will be described in the following subsections.

### 3.1. Asynchronous Interface

Since the frequency and phase of bio-signals from the ADC are different from the MCU circuit, a novel asynchronous interface circuit was integrated into the proposed MCU design. The asynchronous circuit has the potential specifications of low-power consumption, design reuse, improved noise immunity, and high electromagnetic compatibility. One of the most important benefits of the asynchronous circuit is that its power consumption is about 70% less than that of the synchronous design, since the latter tends to draw a large amount of current at the clock edge and shortly thereafter. As shown in Figure 2, the signals with different clock domains can be passed by the asynchronous interface in a wireless body sensor node. The asynchronous interface provides data link services that enable the MCU design to run over a wide range of ADC clock and UART clock frequencies. In the proposed asynchronous interface design, the faster frequency of the operational clock is set as the sampling clock to rebuild the signal from other lower clock domains. Therefore, the signal can access the ADC, MCU, and UART clock domains efficiently and accurately via the proposed asynchronous interface, and prevent the occurrence of handshake problems.

### 3.2. Power Management

**Figure 3.**
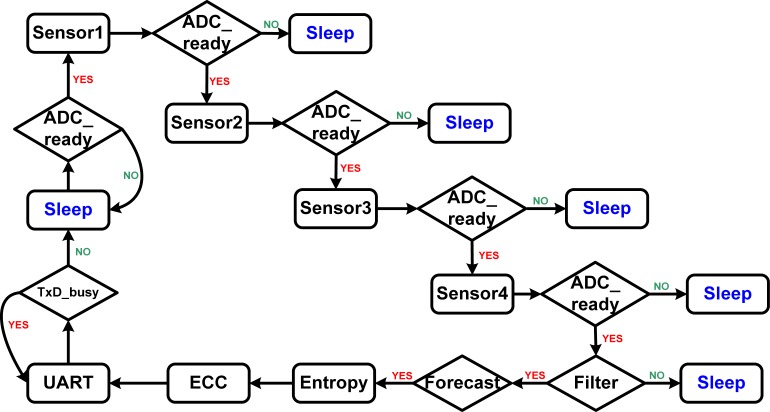
PWM control flow for the wireless sensor node.

**Figure 4.**
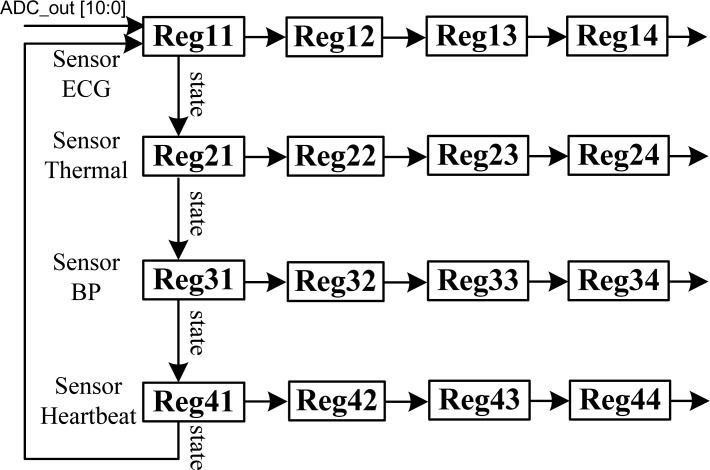
Architecture of the register bank

As the transmitting device contributes most of the power consumption of the WBSN system, an innovative power management controller was created to reduce the transmission power intelligently of the system. The power management method automatically changes the mode from Active to Sleep for each of the devices in the wireless body sensor node. Figure 3 shows the power management control flow for the wireless body sensor node. The control flow contains nested conditional operations with several mutually exclusive conditional paths, which can be implemented by a finite state machine (FSM) circuit. As shown in Figure 4, the ADC_ready signal is a control signal for handshaking, which provides the ready information of the output digital data produced by the ADC. The system is initialized by the detected data of sensor 1, and then checks whether the ADC_ready is available or not. If the ADC_ready signal is obtained from the system, the current state is changed to the Sleep state. The Sleep state is used to power off all the unused circuits, which include the sensors, ADC, RF module, and the functions of the MCU. The system will be woken up when it detects the available ADC_ready signal from the ADC. Each function is powered on only when it needs to be used. Furthermore, the task of the central controller is not only to produce control signals for multi-sensor selection and data compression, but also to send power control signals for each part of the system. The proposed power management circuit design can thus reduce power consumption of the wireless body sensor network system efficiently.

### 3.3. Multi-Sensor Controller

A novel multi-sensor controller was generated for the proposed MCU design as this is necessary to support multi-sensor detection. It produces control signals to a multiplexer for signal selection. The multi-sensor controller is operated with a 4-to-1 multiplexer which has a Boolean equation to connect with four input signals, known as sensor1, sensor2, sensor3, and sensor4. The MCU sends instructions to the multi-sensor controller, which then produces control signals so that the multiplexer can select the sensor based on the hierarchy of the operation. If more than two sensors are working simultaneously, the data from different sensors can be passed-through correctly. Each sensor has its own buffer for storing the data obtained through the multiplexer’s selection procedure. The sensor-select enable signal can make sure that the output data is processed independently. Therefore, the MCU has the ability to prevent the data stream from having missing data based on the design of the multi-sensor controller.

### 3.4. Register Bank

The proposed MCU design includes a reconfigurable filter, so the register bank was added as a novel partition. The register bank was designed for storing a collection of values and the position of each value cloud be computed from its index mark by a mathematical formula. Register banks are widely used in signal processing, as the element indices can be obtained by real-time computing operations. Figure 4 shows the architecture of the register bank used in this work. The input signal of the register bank comes from the ADC, and the strings are stored separately according to different bio-sensors, such as ECG, thermal, blood pressure, and heartbeat. Four data strings are stored in four homogenous registers, and the value of each register would be shifted down to the next register when a new value comes from the ADC. After the signals from the four sensors are stored in the first column of the register bank, the values in each column will be shifted right to the next column. The register bank design can store four continuous values for each sensor signal, and provide these to the digital filter and data encoder for signal processing.

### 3.5. Reconfigurable Filter

The reconfigurable filter is designed to filter various bio-signals by using only one filter circuit. Since the characteristics of bio-signals are very different, the coefficients of different digital filters will also be very different. Moreover, the spectra of different bio-signals are very different, so various filters with different characteristics are used for signal filtering. The filter was designed with a novel reconfigurable architecture, which can be transferred to an average, a binomial, and a sharpen filters immediately. The architecture of the average, binomial, sharpen, and reconfigurable filter will be described in the following subsections:

#### 3.5.1. Average Filter (Low Pass Filter)

**Figure 5.**
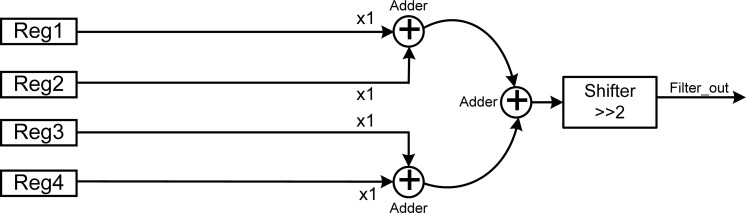
Architecture of the average filter.

The average filter is a kind of low-pass filter, with the characteristic that all of the weight coefficients are the same and the filtered result is the average of the input values. Figure 5 shows the architecture of the average filter which consists of three adders and one shifter. The four input values can be obtained from the register bank as Reg1, Reg2, Reg3, and Reg4, and the values of these can be updated when the new detected value arrives. The values of Reg1 and Reg2 are added by an adder, as well as those of Reg3 and Reg4. The two added results are then added by one more adder to produce the sum of these four values stored in these four registers. Finally, the sum of the four values is divided by four by using the shifter to shift two bits right. The average filter can be implemented by only the adders and shifter without any multiplier and divider, which achieves the benefit of low hardware cost for VLSI implementation.

#### 3.5.2. Binomial Filter (Band Pass Filter)

**Figure 6.**
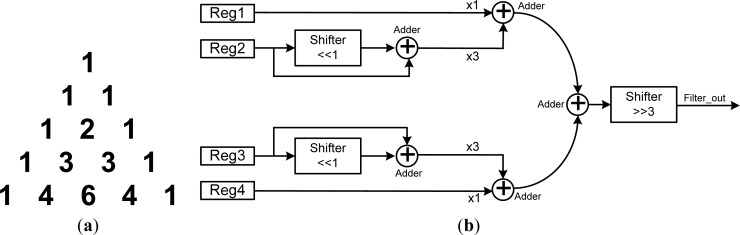
(**a**) Pascal’s triangle. (**b**) Architecture of the proposed binomial filter.

The binomial filter is a kind of band-pass filter [18], in which the binomial coefficients can be formed by a Pascal’s triangle, as shown in Figure 6(a). The binomial coefficients of the proposed binomial filter can be obtained by:

(1) $${\left(1+x\right)}^{n}=\left(\begin{array}{c} n\\ 0 \end{array}\right)x^{0}+\left(\begin{array}{c} n\\ 1 \end{array}\right)x^{1}+\left(\begin{array}{c} n\\ 2 \end{array}\right)x^{2}+\cdots +\left(\begin{array}{c} n\\ n-1 \end{array}\right)x^{n-1}+\left(\begin{array}{c} n\\ n \end{array}\right)x^{n}$$

where $\left(\begin{array}{c} n\\ k \end{array}\right)$ is binomial coefficient for each power of *x*, and *k* and *n* indicate the number of elements and the size of the element set. If *n* is assigned equal to 3, for example, the binomial coefficients will be 1, 3, 3, and 1, which correspond to the fourth row of Pascal’s triangle, as shown in Figure 6(a). Figure 6(b) shows the architecture of the proposed binomial filter. In order to reduce the hardware cost, the binomial filter is implemented by five adders and three shifters. The original value times of coefficient 3 can calculated by using only one shifter and one adder, which can be greatly reduce hardware cost by avoiding the use of a multiplier. The result of the proposed binomial filter can be calculated by shifting three bits right from the sum of the weights.

#### 3.5.3. Sharpen Filter (High Pass Filter)

**Figure 7.**
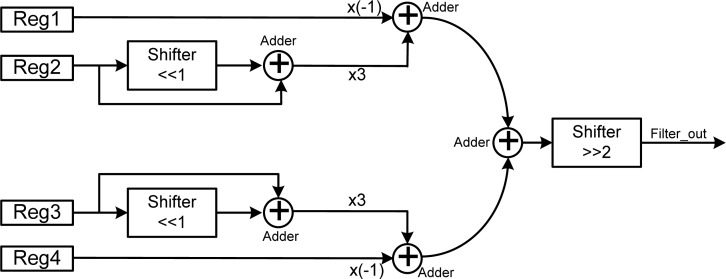
Architecture of the proposed sharpen filter.

The sharpen filter is a kind of high-pass filter, which can pass high frequency signals but cutoff signals with low frequency. A wide variety of alternative leaner sharpen filters can be used to enhance high frequency details and reduce low frequency noise. The proposed sharpen filter is based on the Gaussian [19] equation defined by:

(2) $$G(x)=\frac{1}{\sqrt{2\pi \sigma^{2}}}e^{\left(-\frac{x^{2}}{2\sigma^{2}}\right)}$$

where *x* is the distance from the original signal and σ is the standard deviation of the Gaussian distribution. Values from this distribution are used to build a convolution signal which is applied to the original data. Figure 7 shows the architecture of the proposed sharpen filter.

To reduce the hardware cost, the proposed sharpen filter is designed by using only four adders and three shifters. The coefficients of the proposed sharpen filter are −1, 3, 3, and −1, which corresponds to enhance the center of the signals and remove the influence of neighboring ones. The proposed sharpen filter enhances the details of high-frequency signals and removes other details of low-frequency signals present in noise, and thus provides an efficient resolution of high-pass filtering the bio-signals.

#### 3.5.4. Reconfigurable Filter

**Figure 8.**
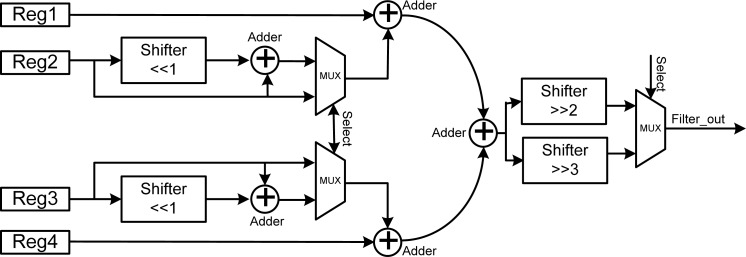
Architecture of the proposed reconfigurable filter.

The three kinds of filter mentioned above provide different spectra for filtering various bio-signals, and can thus provide a complete filter set for wireless multi-sensor network systems. However, implementing these three kinds of filters requires a lot of chip area, and only one filter can be used during each detection time. Therefore, this work proposes a reconfigurable filter to replace the average, binomial, sharpen, and reconfigurable ones. The principle behind this is to use control signals to control the behavior of the reconfigurable hardware. Therefore, the main concept in the design of the proposed reconfigurable filter is using three multiplexers and one control signal to configurable the reconfigurable filter as an average, binomial or sharpen one. Figure 8 shows the architecture of the proposed reconfigurable filter, which consists of five adders, four registers, and three multiplexers.

Table 1 lists the hardware cost of the average, binomial, sharpen, and the proposed reconfigurable filters. The average filter consists of two adders and one shifter, and the equivalent gate count is 2.53 K-gates. The binomial filter is a little more complex than the average filter. It is composed of five adders and three shifters, and the cost is about 2.69 K-gates. The sharpen filter is composed of five adders and three shifters, and its gate count is almost the same as that of the binomial filter. The total hardware costs of the average, binomial, and sharpen filters are twelve adders and seven shifters, which equals 7.92 k-gates. However, the proposed novel reconfigurable design costs only five adders and four shifters, which equals 3.24 K-gates. The proposed novel reconfigurable filter design provides the same functionality while reducing the adders by 58.3%, shifters by 42.8%, and gate counts by 59%. It provides a novel low-cost reconfigurable architecture for developing three different kinds of filters for wireless multi-sensor network systems.

### 3.6. Lossless Data Encoder

The lossless data encoder consists of three parts: a slope-feature forecaster, an entropy encoder, and an error correct coding (ECC) encoder. In this work, the slope-feature forecaster and entropy encoder can be integrated as a lossless data compression encoder. To improve the error bit rate from wireless transmission, an error correcting code encoder is used. The details of each part of the system are described in the following subsections:

#### 3.6.1. Slope-Feature Forecaster

**Figure 9.**
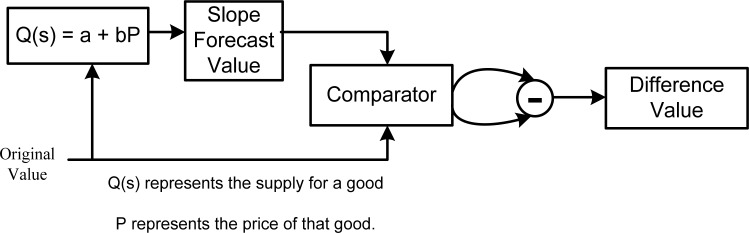
The architecture of the slop feature forecaster.

With the four signal values stored in the register bank, the latest of the two values can be used to operate the newest value in predicatively. The methodology of the proposed forecaster design is based on slope prediction, as shown in Equation (3):

(3) Q(s)=a+bP

where *a* is a positive constant, *b* is the value of the slope, and the *P* is the latest value of the original signals. Therefore, the response of the equation *Q(s)* is the result of the forecasting value. Figure 9 shows the architecture of the slope-feature forecaster.

The input values of the slope forecasting step can be obtained from the register bank, and then the forecast value is processed and compared with the original one. After comparison by the comparator, a subtract circuit is used to calculate the difference between forecast value and the original one, with the result called the difference value. Finally, the difference value and sign information are produced and then send to the entropy encoder as input. One common example is the variation of ECG, which is generally very large, and the difference value between two points can reach several hundreds of units by an 11-bits ADC. Using the slope forecast design, the probabilities can be concentrated within a small range of difference values. On the other hand, if the biomedical signals contain fewer variations, as seen with body temperature, for example, the probabilities are gathered and the difference values are distributed around zero. The proposed novel slop feature forecast consists of a comparator and one sub-tractor, which costs a small chip area but improves the compression rate efficiently.

#### 3.6.2. Entropy Data Encoder

The entropy encoder is a lossless data compression circuit based on Huffman coding [20]. The principle of entropy coding is based on creating and assigning a unique prefix-free code to each unique value produced by the slope forecaster. After receiving the difference value and sign information from the slope forecaster, the entropy encoder replaces each 11-bit input difference value and 1-bit sign information with the corresponding variable length code (VLC). The length of each code is approximately proportional to the negative logarithm of the probability. Therefore, the most common values are replaced by the shortest variable length codes. The term refers to the use of the variable length code chart for encoding the difference values, and the chart has been derived in a particular way is based on the estimated probability of occurrence for each possible value of the difference values.

**Figure 10.**
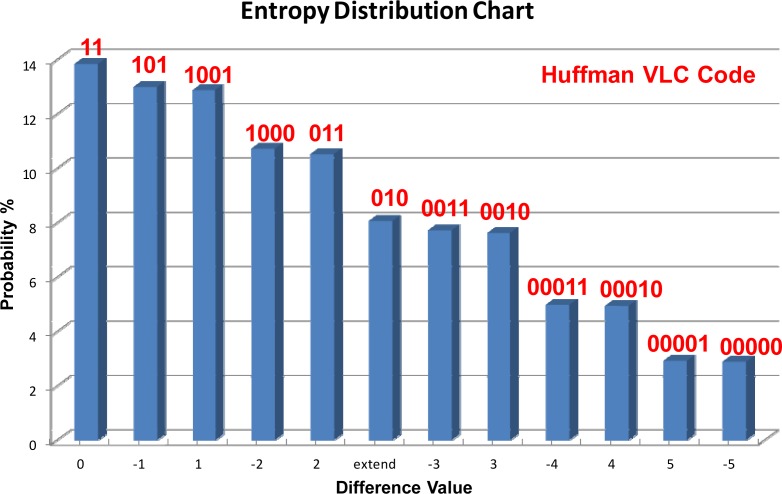
The probability distribution and VLC of the proposed entropy data encoder.

The Huffman algorithm considers both the probability and length of code. The basic concept behind Huffman coding is to represent more frequent data by shorter codes, and less frequent data by longer ones. Therefore, the average code length is expected to be shorter than that of the fixed-length representation. The probability distribution and variable length codes of the proposed entropy data encoder is shown in Figure 10. To analyze the performance of the proposed slope forecast method and entropy encoding skills, the MIT-BIH Arrhythmia database [23] for ECG signals is used for testing. The ECG signals were digitized by sampling at 360 samples per second, and then quantized and encoded into eleven bits per sample. In this paper, the MIT-BIH database is used as an example to obtain the compression rate of the data encoder. The average result of the compression rate (CR) is over 2.53, which means that more than half the ECG data rates are reduced by the proposed data encoder design.

#### 3.6.3. Error Correct Coding (ECC) Encoder

The error correct code (ECC) is a technique used to control the errors that can occur during data transmission due to unreliable or noisy wireless communication channels. The principle of the ECC involves adding additional information, called redundancy codes, into the original data for transmission. After receiving the encoded data from the transmitter, the receiver can check the correctness of the received data by using the ECC decoder. If the decoder detects a limited number of errors that may occur anywhere in the message, the errors can be corrected by the redundancy codes without requiring retransmission. The ECC decoder can check the received data with a cyclic redundancy technique, which is a single burst error detecting cyclic code designed to detect transmission errors. This applies a generation polynomial, which is used as the divisor in polynomial long division over a finite field. The ECC decoder can automatically check the received stream, and if it is correct, then it sends out the data directly. On the other hand, if the received stream is incorrect, the ECC decoder corrects the error bits immediately and then send out the corrected data. This error correcting coding design improves the reliability of biomedical signals for the WBSN systems.

### 3.7. Universal Asynchronous Receiver/Transmitter (UART) Interface

**Figure 11.**
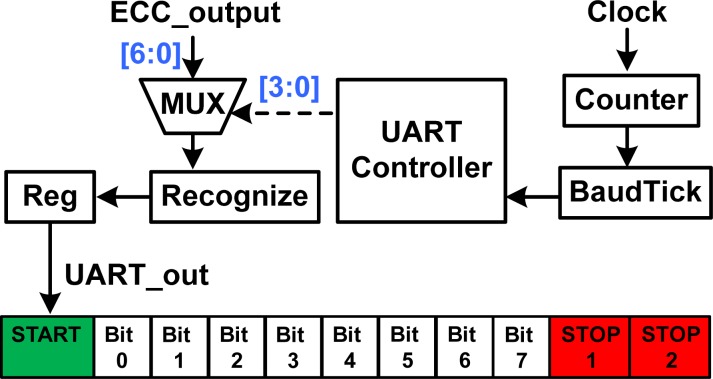
Architecture of the proposed UART interface design.

The UART interface [21] is commonly used in conjunction with communication standard protocols, such as RS232. The RS232 transceiver is a peripheral device used with computers, and can transmit data in serial forms. Figure 11 shows the architecture of the proposed UART interface design. The UART interface is controlled by a UART controller that can produce control signals to select one bit of the ECC_output data as one bit of the output signal UART_out. As soon as the data is deposited in the shift register after completion of the previous data, the UART interface will generate the output signal UART_out including one bit of start information, eight bits of data information from bit0 to bit7, and two bits of stop information. Since transmission of a single character may take a long time, the UART interface will maintain a flag related to the baud rate to show busy information. Hence, the UART controller will keep the current transmission status until the previous transmission is finished. The UART interface can be designed with the ECC encoder, and then the uncertain error bits that are received can be corrected by the ECC decoder. Therefore, the UART interface design improves the compatibility of the proposed MCU design with PCs or wireless devices.

## 4. Simulation Results and Chip Implementation

**Figure 12.**
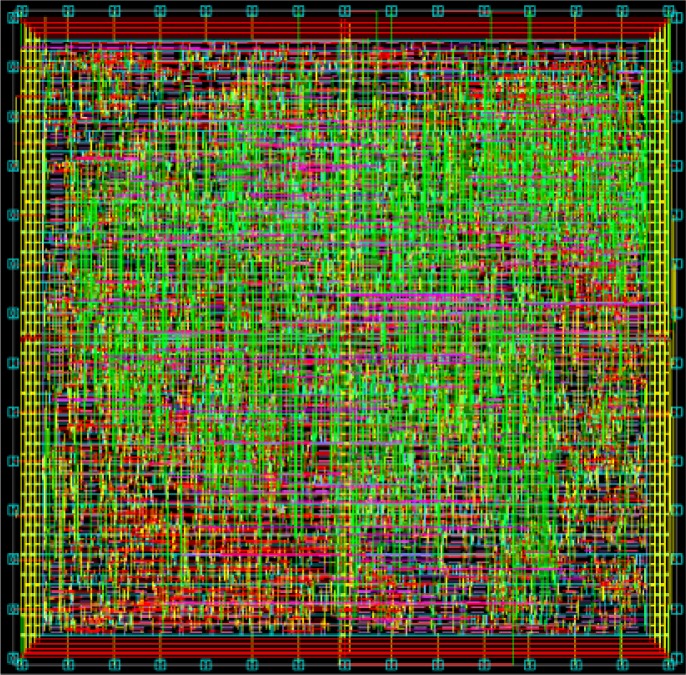
The Layout in TSMC 0.18 μm Process.

To realize the proposed design, in this work the Verilog hardware description language was used to implement the VLSI architecture. The Design Compiler, an electronic design automation (EDA) tool, was used to synthesize the proposed MCU design based on TSMC 0.18 μm and 0.13 μm CMOS generic logic process technology. The layout of this work was generated by the auto place and route (APR) tool IC Compiler, and Figure 12 shows the photomicrograph of the chip used in this work. The core area of this work is 0.076-mm^2^ or 0.037-mm^2^, and it consumes 5.8 mW or 1.9 mW when operating at 100 MHz or 133 MHz frequency with the TSMC 0.18 μm or 0.13 μm CMOS process, respectively.

**Figure 13.**
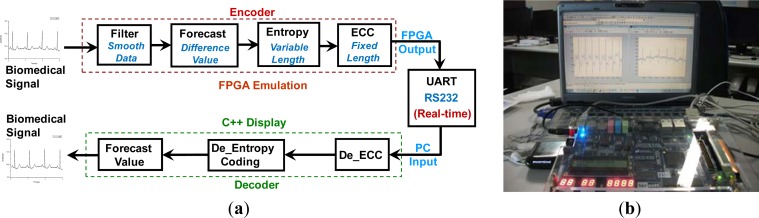
The emulation flow and environment of the proposed design (**a**) Block diagram; (**b**) Prototype system.

Figure 13(a) shows the emulation flow of the proposed design. It consists of an Altera EP4C115F29C7N FPGA core, a transmission interface (RS232 protocol), and a software system. Figure 13(b) shows a system prototype implementation, which includes a FPGA verification board, RS232 interface, and a personal computer.

First, the biomedical signals are filtered by the reconfigurable filter for high-pass, low-pass, or band-pass filtering, which can be set by users according to the characteristics of the biomedical signals. Next, the filtered signals are passed to the slope-feature forecast circuit and the difference values extracted from the original data can be obtained. After forecasting, the difference values are sent to the entropy encoder based on the Huffman coding algorithm, and then encoded by the ECC encoder to increase the reliability of the wireless device based on the UART protocol. The encoded stream data is then be sent to PC by the wireless UART device and refined by the decoding processes which were developed using C language and executed by a PC. Finally, the refined biomedical signals are compared with the original ones to make sure all the functions of the proposed MCU design are correct.

Table 2 lists the comparison results of the number of sensors, process, function, operation frequency, gate count, power consumption, and chip area of six efficient MCU designs with this work. As compared with six previous designs, this work achieves supporting four various biomedical sensors, which is four times of previous works [9–13] and the same as our previous work [8]. To be able to compare with the previous works [8,9,11,12], this work was synthesized by 0.18-μm and 0.13-μm processes. The functions in this work includes the slop compression, power management (PWM), asynchronous interface, ECC, register bank, reconfigurable filter, and UART interface, which are much more than previous designs [8–13]. The gate count of this work is 7.67-K which is much less than other low-cost MCU cores [9–13]. The power consumption of this work is 5.8 mW or 1.9 mW operating at 100 MHz or 133 MHz with TSMC 0.18-μm or 0.13-μm process.

Comparing the results with those in our previous work [8], although the chip area and power consumption in the current study are both higher, the functions of slope compression, register bank, reconfigurable filter, and UART interface are all better. Table 3 shows the gate count of each function in the proposed MCU design. The gate count of the multiple selector auto-detecting four sensors is 1.88 K-gates. The reconfigurable filter and register bank are implemented by the most gates in the proposed MCU design, 3.24 K, and can provide the functions of low-pass, high-pass, and band-pass filters for the WBSN system. The lossless data compression circuit based on the Huffman encoding algorithm and the error correct coding circuit (ECC) are realized by 3.77 K-gates and 1.68 K-gates. The PWM circuit is designed for the purpose of saving power, and can be realized by a finite state machine (FSM) with only 0.12 K-gates. The UART interface, which is used to increase the compatibility with wireless devices for communication transmission, costs 0.44 K-gates.

Table 4 lists the comparison rates in our previous work [8] and the current one based on patterns in the MIT-BIH ECG database [23] with the numbers 112, 115, 121, 201, 205, and 231. The compression rate of this work for pattern 112 is 2.59, which is much better than the 2.35 obtained in our previous work. It is clear that the compression rates in this work improve on those in the earlier work by between 2.02% to 13.27%, and this is due to the slope-feature forecaster design. The compression rate of MIT-BIH Arrhythmia data in this work is 2.38.

## 5. Conclusions

A multiple-function micro control unit (MCU) design for wireless body multi-sensor networks is presented in this paper. It improves on earlier designs by adding the slope-feature forecaster, register bank, reconfigurable filter, and UART interface to enhance the compression rates, remove signal noise, and increase the compatibility for the MCU design. The proposed MCU design can operate at 133 MHz with a power consumption of 1.9 mW. Compared with previous designs, this one has the benefits of using less power and offering multiple sensor support, as well as less noise, a lower transmission error rate, and a lower data rate. These characteristics provide an efficient basis for developing wireless body sensor network (WBSN) systems.

## Figures and Tables

**Table 1.** Hardware cost of the average, binomial, sharpen and the proposed reconfigurable filters.

**Filter**	**Adder**	**Shifter**	**Gate Count (including Register-Bank)**
**Average Filter (Low-Pass)**	2	1	2.53 K
**Binomial Filter (Band-Pass)**	5	3	2.69 K
**Sharpen Filter (High-Pass)**	5	3	2.70 K
**Total Filters (Average + Binomial + Sharpen)**	**12**	7	**7.92 K**
**Reconfigurable Filter (This Work)**	**5**	4	**3.24 K**

**Table 2.** Comparison of previous MCU designs with this the one presented in this work.

	**Sensors No.**	**Process**	**Function**	**Operating Freq. (Hz)**	**Gate Count**	**Power (W at Hz)**	**Chip Area (mm^2^)**
[13] JSSC ‘04	1	0.60 μm	Low-Pass Filter	1.2 K	190 K	560 n at 1.2 K	3.200
[10] ICASSSP ‘04	1	0.50 μm	RAM, ROM	8.2 M	Non	60 m at 8.2 M	None
[12] IEICE ‘08	1	0.18 μm	Filter, Compression, Calibrator	100 M	Non	631 μ at 100 M	<1
[9] ISJ ‘09	1	0.18 μm	Compression, PWM	100 M	13.4 K	150 μ at 1 M	0.134
[11] JSSC ‘09	1	0.13 μm	MUX, ALU, RAM, DMA	75 M	110 K	120 μ at 75 M	0.430
[8] Sensors ‘11	4	0.13 μm	Compression, PWM, Asynchronous, ECC	133 M	2.68 K	496 μ at 133 M	0.014
This work	4	0.18 μm 0.13 μm	Slope Compression, PWM, Asynchronous, ECC, Register Bank, Reconfigurable Filter, UART interface	100 M 133 M	7.67 K	5.8 m at 100 M 1.9 m at 133 M	0.076 0.037

**Table 3.** The Gate Count of each Function in MCU design.

**Function**	**Multiple Selector**	**Register Bank & Reconfigurable Filter**	**Lossless Compressor (Forecaster + Entropy)**	**ECC**	**PWM**	**UART**
**Gate Count**	1.88 K	3.24 K	3.77 K	1.6 K	0.12 K	0.44 K

**Table 4.** Comparison of the Data Compression Rates.

**MIT-BIH Pattern No.**	**Original (KB)**	**Compression Rate (CR) of Previous Work [**8**]**	**Compression Rate (CR) of this Work**

112	1,696	2.35	2.59
115	1,696	2.48	2.53
121	1,696	2.26	2.56
201	1,696	2.45	2.53
205	1,696	2.51	2.68
231	1,696	2.41	2.56
